# Identification of a small‐molecule ligand of *β*‐arrestin1 as an inhibitor of stromal fibroblast cell migration accelerated by cancer cells

**DOI:** 10.1002/cam4.1339

**Published:** 2018-01-29

**Authors:** Kruthi Suvarna, Kaori Honda, Yasumitsu Kondoh, Hiroyuki Osada, Nobumoto Watanabe

**Affiliations:** ^1^ Bio‐Active Compounds Discovery Research Unit RIKEN Center for Sustainable Resource Science Saitama Japan; ^2^ Tokyo Medical and Dental University Tokyo Japan; ^3^ Chemical Biology Research Group RIKEN Center for Sustainable Resource Science Saitama Japan

**Keywords:** Fibroblasts, ligands, signal transduction, tumor microenvironment, wound healing

## Abstract

Stromal fibroblasts, which occupy a major portion of the tumor microenvironment, play an important role in cancer metastasis. Thus, targeting of these fibroblasts activated by cancer cells (carcinoma‐associated fibroblasts; CAFs) might aid in the improved treatment of cancer metastasis. NIH3T3 fibroblasts cocultured with MCF7 cells displayed enhanced migration compared to NIH3T3 fibroblasts cultured alone. We used this system to identify the small‐molecule inhibitors responsible for their enhanced migration, a characteristic of CAFs. We selected *β*‐arrestin1, which showed high expression in cocultured cells, as a molecular target for such inhibitors. Cofilin, a protein downstream of *β*‐arrestin1, is activated/dephosphorylated in this condition. The small‐molecule ligands of *β*‐arrestin1 obtained by chemical array were then examined using a wound healing coculture assay. RKN5755 was identified as a selective inhibitor of activated fibroblasts. RKN5755 inhibited the enhanced migration of fibroblasts cocultured with cancer cells by binding to *β*‐arrestin1 and interfering with *β*‐arrestin1‐mediated cofilin signaling pathways. Therefore, these results demonstrate the role of *β*‐arrestin1 in the activation of fibroblasts and inhibiting this protein by small molecule inhibitor might be a potential therapeutic target for the stromal fibroblast activation (cancer–stroma interaction).

## Introduction

Treatment of metastatic breast cancer, which is often incurable, faces the challenge of the tumor microenvironment. A number of studies examining tumor microenvironments have made it clear that fibroblasts, which comprise a major component of cancerous stroma, play an important role in cancer metastasis 1. The cross‐talk that occurs between stromal fibroblasts and cancer cells leads to morphological changes in normal fibroblastic cells. Fibroblasts that are activated by cancer cells are called carcinoma‐associated fibroblasts (CAFs), and they are known to guide cancer cell migration by mechanically modifying tumor stroma 2. Recent studies have described how the interaction of CAFs with the cancer cells can lead to chemotherapy resistance 3, 4. Thus, a combination of conventional therapy, along with CAF‐directed therapy, might lead to improved treatment of breast cancer metastasis.

The major challenge in targeting CAFs is their heterogeneity. Although numerous studies have been performed on CAFs, a specific marker recognizing these cells is lacking. It has long been known that fibroblasts become activated during wound healing, and cancer has been described as a wound that does not heal or as an over healing wound 5, 6. The link between fibrosis and cancer suggests a similar phenomenon in activated fibroblasts migrating faster than normal fibroblasts. Ishii et al. 7 identified enhanced migration as one of the biological properties of CAFs. Studies on the role of CAFs in cancer cell migration and invasion are already documented 8; however, the mechanism of fibroblast activation by cancer cells is still unclear.

*β*‐arrestins were first discovered as a protein involved in desensitization of G‐protein‐coupled receptors (GPCRs), and it is now clear that it acts as an adaptor molecule that binds to a number of effector molecules, thus modulating multiple GPCR‐dependent and GPCR‐independent signaling pathways 9. *β*‐arrestins are reported to be expressed in stromal cells 10. Recent studies have also shown the involvement of *β*‐arrestins in fibrotic diseases, acting as a mediator of fibroblast invasion and thus a potential target for treatment of fibrosis 11. Several studies showed that knockout of *β*‐arrestin in mouse embryonic fibroblast cells decreases cell migration, thereby revealing a novel role for *β*‐arrestin in maintaining cell motility and regulating cell shape 12.

There are a number of factors involved in activation of fibroblasts, including growth factors, enzymes, cytokines, and tumor suppressors. Among cytokines, chemokines CXCL14, and CXCL12 play an important role in paracrine cross‐talk between fibroblasts and cancer cells. CXCR4/CXCR7 heterodimeric chemokine receptors activated by these chemokines recruit *β*‐arrestin1, which enhances cell migration 13, 14.

Thus, *β*‐arrestin might play an important role in activating stromal fibroblasts present in the vicinity of cancer cells. Recently, a role for *β*‐arrestin in activation of the actin filament‐severing protein cofilin was reported 15. Cofilin plays an important role in the formation of membrane protrusions and cell migration. *β*‐arrestin is an adaptor protein that binds to cofilin, LIM kinase (which inactivates cofilin by phosphorylation on ser^3^), and other cofilin‐specific phosphatases such as chronophin and slingshot, which dephosphorylates and activates it. The cofilin phosphatase activity is regulated mainly by *β*‐arrestin1. *β*‐arrestin is also involved in localization of cofilin to membrane protrusions thus creating a new barbed end for elongation and pseudopodia formation 15, 16. Therefore, inhibition of *β*‐arrestin‐depending signaling pathways involved in actin polymerization might be one way to target fibroblast activation by cancer cells.

In this study, we established a wound healing assay system using coculture of NIH3T3 mouse embryo fibroblast cell line and MCF7 breast cancer cells and used this system to identify a novel compound that specifically targets CAFs. To identify a compound that could slow down the accelerated migration of fibroblast cells, we focused on *β*‐arrestin1 and tried to obtain its small‐molecule ligands through chemical array‐based high‐throughput screening. Using a wound healing coculture assay for secondary screening, we identified RKN5755 as a selective inhibitor of activation of fibroblasts by cancer cells. In addition, we examined the mechanism by which the compound inhibits accelerated migration via interference with the *β*‐arrestin1–cofilin scaffolding pathway.

## Materials and Methods

### Materials

Compound RKN5755 was obtained from RIKEN Natural Products Depository (NPDepo, Saitama, Japan). The compound was dissolved in dimethylsulfoxide (DMSO) as a stock solution, which was stored at −20°C.

### Cell culture

The mouse embryonic fibroblast cell line NIH3T3, the human lung fibroblast cell line WI‐38, the human breast cancer cell line MCF7, the human cervical cancer cell line HeLa, and the human lung cancer cell line A549 (RIKEN Cell Bank) were cultured in D‐MEM (Invitrogen), supplemented with 10% fetal calf serum (FCS) (Nichirei Bioscience Inc. Tokyo, Japan), 50 U/mL penicillin (Invitrogen), and 50 *μ*g/mL streptomycin (Invitrogen), at 37°C in a humidified atmosphere containing 5% CO_2_.

### Green fluorescence protein (GFP) labeling

MCF7 cells were transfected with pEGFP‐N1 vector (Clontech) encoding EGFP using the Effectene transfection reagent kit according to the manufacturer's instructions (Qiagen, Cat.No. 301425). Stably transfected GFP‐positive MCF7 cells (MCF7^GFP^) were selected using G418 (1000 *μ*g/mL) and were maintained at 500 *μ*g/mL (Calbiochem, Cat.No. 345812).

### Direct coculture wound healing assay

NIH3T3 fibroblast cells (2 × 10^5^) were seeded on a 6‐well plate at 80% confluency. For coculture experiments, MCF7^GFP^ cells (0.4 × 10^5^) were seeded on top of fibroblast cells 2 h after seeding NIH3T3 cells. After incubating the cells overnight in DMEM + 10% FCS, a scratch was created using a 200‐*μ*L tip. The cells were then washed twice with 1% PBS, and the medium was changed to DMEM + 1% FCS with or without the indicated dose of the compound. At each interval, the cells were photographed using an inverted fluorescent microscope (Olympus IX70), and NIH ImageJ software U. S. National Institutes of Health, Bethesda, Maryland, USA was used for quantification of the scratch wound assay. Protein lysates from plates with NIH3T3 alone (2 × 10^5^) cells and MCF7 cells (2 × 10^5^) cultured alone were mixed at 5:1 ratio, quantified, and used as a control for cocultured sample in Western blotting analysis.

### Cell proliferation assay

NIH3T3 and MCF7 cells were seeded in a 96‐well culture plate and cultured overnight in 100 *μ*L DMEM + 10% FCS. On the next day, the medium was changed to DMEM + 1% FCS with or without the indicated dose of the compound. After 48 h, cell growth was measured using the Cell Count Reagent SF (Nacalai Tesque) according to the manufacturer's instructions. Briefly, 10 *μ*L of WST‐8 solution was added to each well, and the plates were incubated at 37°C for 2 h. Then, cell growth was measured as the absorbance at 450 nm on a microplate reader (Perkin Elmer).

### Transwell migration assay

The migration assay was performed using 24‐well culture chambers (Iwaki microplate) and a polycarbonate filter with an 8 *μ*m pore size (Kurabo, Chemotaxicell). The NIH3T3 fibroblast cells (2 × 10^5^), cocultured NIH3T3: MCF7 cells (5:1; 2 × 10^5^ + 0.4 × 10^5^), and MCF7 cells (2 × 10^5^) were serum‐starved using DMEM + 1% FCS. After overnight starvation of cells, 5 × 10^4^ NIH3T3 cells, cocultured cells, and MCF7 cells were seeded into the upper chamber containing 200 *μ*L DMEM medium devoid of serum. The lower compartment contained 600 *μ*L of DMEM + 10% FCS. Indicated doses of the compound were added to both chambers 17. Seeding was performed in triplicates, and the serum‐starved cells were allowed to migrate toward the serum‐containing medium for 12 h at 37°C in a humidified incubator under a 5% CO_2_ atmosphere. At the end of the incubation period, the cells that passed through the filter into lower wells were fixed with 3.7% formaldehyde and were stained with 1% crystal violet. The cells in three predetermined fields were counted under an inverted microscope.

### Purification of *β*‐arrestin1

GST‐*β*‐arrestin1 from human in a pGEX‐5X‐1 vector 18 was transformed into *E.coli* BL21 star (DE3) cells. The bacteria were grown on LB media and induced for protein expression using 0.4 mmol/L IPTG for 15 h at 20°C. They were then pelleted and lyzed using sonication in lysis buffer (0.5% Tween‐20, 150 mmol/L NaCl, 1 mmol/L EDTA, pH 8.0). The GST‐*β*‐arrestin1 fusion protein was purified using the glutathione sepharose 4B (GE Healthcare), then quantified and used for the assay.

### Chemical array screening

The chemical arrays were prepared by previously described method 19, 20. We used 29,707 compounds from NPDepo for the chemical arrays. Chemical array screening was carried out as previous described 20. The concentration of GST‐*β*‐arrestin1 was 1 *μ*mol/L when applied to the chemical arrays.

### Western blotting

Western blot analysis was performed as follows. The cells were lyzed in whole‐cell extraction buffer (1% Triton X‐100, 0.5% sodium deoxycholate, 0.1% SDS, 150 mmol/L NaCl, 1 mmol/L EDTA, 10 mmol/L NaF, 1 mmol/L sodium orthovanadate, 2 mmol/L sodium pyrophosphate, and 50 mmol/L Tris‐Cl pH 8.0) containing a complete protease inhibitor cocktail tablet (Roche Diagnostic, Mannheim, Germany). Next, 20 *μ*g of cleared lysate protein was analyzed by SDS‐PAGE (10% gel for *β*‐arrestin1 and 15% gel for cofilin) and then transferred to an Immobilon‐P PVDF (polyvinylidene fluoride) membrane (Millipore, Billerica, M.A). The blots were saturated with blocking buffer (5% skim milk in TBS‐T) for 1 h at room temperature and incubated with the appropriate antibodies. The antibodies and final dilutions used for Western blot (WB) analysis were as follow: rabbit anti‐*β*‐arrestin1 monoclonal antibody specific for both human and mouse protein from Abcam [E274, ab32099] (1:1000), rabbit anti‐phospho(ser^3^)‐cofilin polyclonal antibody specific for both human and mouse protein from Santa Cruz Biotechnology [SC‐21867‐R] (1:1000), and rabbit anti‐cofilin monoclonal antibody specific for both human and mouse protein from Cell Signaling Technology [5157] (1:1000). After washing in TBS‐T, the membrane was incubated for 2 h at room temperature with HRP‐donkey anti‐rabbit IgG (GE healthcare). Antibody binding was detected with the SuperSignal West Pico chemiluminescent substrate (Thermo Scientific Massachusetts, United States).

### Pull down assay

RKN5755 beads were prepared as previously described 21. Purified GST‐*β*‐arrestin1 fusion protein was incubated with RKN5755 beads for 3 h. The beads were then washed in 1 mL of binding buffer and eluted with SDS‐PAGE sample buffer. The eluted protein was then subjected to SDS‐PAGE and stained with Coomassie brilliant blue (CBB).

### Statistical analysis

All experiments were performed at least three times. Data are expressed as mean ± SD. Statistical comparison between two groups was made by student's *t* tests. Tukey–Kramer method was used for multiple comparisons. Values of *P* < 0.05 were considered significant (*), *P* < 0.005 were considered very significant (**), and *P* < 0.0005 were considered extremely significant (***).

## Results

### Migration of fibroblast cells was enhanced when cancer cells were cocultured

**Figure 1:**
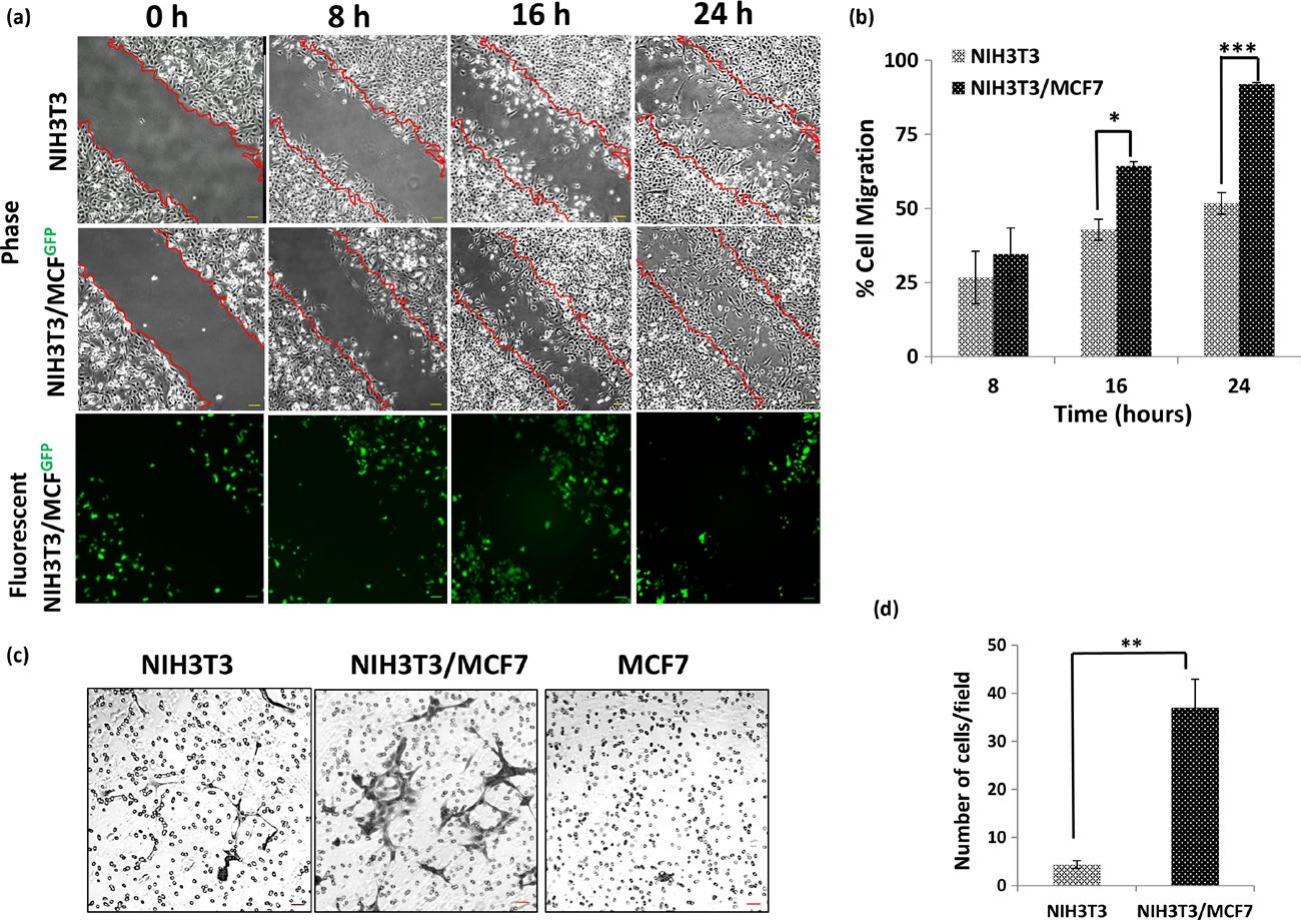
NIH3T3 cells cocultured with MCF7^GFP^ cells display enhanced migration activity compared to culture of NIH3T3 cells alone. (A) NIH3T3 cells alone and NIH3T3 cells cocultured with MCF7 were seeded in 6‐well plates, scratched, and cultured in medium containing 1% FCS. Cell migration was observed at different time points until 24 h. The edge of the wound at 0 h was marked with a red outline and is shown overlaid with other images at different time points. Images of MCF7 tagged with GFP in coculture were taken by fluorescent and light microscopy. (B) The cell migration percentage for fibroblasts and cocultured cells at different time points was quantified using ImageJ software. There was a significant difference in the migration of cocultured fibroblasts when compared to culture of fibroblasts alone at 16 and 24 h (*n* = 3,*P < 0.05, ***P < 0.0005). (C) NIH3T3 cells and/or MCF7 cells were cocultured for 24 h in medium containing 1% serum and transferred into the upper chamber of a transwell system without serum and allowed to migrate toward the bottom chamber with 10% serum levels. The membrane was stained with crystal violet after 24 h of migration, and images of the three different fields were captured with an inverted microscope. (D) The number of fibroblast cells and cocultured cells that migrated toward the lower side of the membrane was quantified by ImageJ. NIH3T3 cell migration was significantly higher when cocultured compared to the migration of NIH3T3 cells alone. (*n* = 3, **P < 0.005).

We examined the migration of fibroblast cells by a traditional wound healing assay. NIH3T3 cells were seeded, scratched, and cultured in the medium containing 1% FCS to prevent further cell growth. Migration of cells was detected as early as 8 h after scratch formation and was quantitated using imaging software (Fig. 1A and B). To examine the effect of cocultured cancer cells on the migration of fibroblasts, low‐invasive MCF7 breast cancer cells were mixed with NIH3T3 cells, and the migrations of cells were examined. As expected, the migration of NIH3T3 cells was significantly enhanced in the presence of MCF7, occurring as early as 16 h after scratch formation (Fig. 1B). We examined several mixing ratios and found that NIH3T3 cell migration was most enhanced when the mixing number of MCF7 cells was one‐fifth that of NIH3T3 (data not shown). As MCF7 are low‐invasive cancer cells, most of the migrated cells were NIH3T3 cells, as determined from the shape of the cells. We also confirmed it using MCF7^GFP^ cells, in which GFP is stably expressed. In coculturing experiments with NIH3T3 and MCF7^GFP^ cells, almost all of the migrated cells were GFP negative, indicating that most of the migrated cells were NIH3T3 cells (Fig. 1A).

The enhanced migration of NIH3T3 cells after cocultured with MCF7 was also detected in the transwell migration assay system. NIH3T3 cells and/or MCF7 cells were (co)cultured for 24 h under serum‐starved conditions. They were then transferred into the upper chamber of the transwell assay system with culture medium lacking serum, and the upper chamber was placed onto the lower chamber containing culture medium with 10% FCS and cultured for 24 h. As shown in Figure 1C and D almost no NIH3T3 or MCF7 cells cultured alone migrated through the filter, but significant numbers of migrated NIH3T3 cells could be detected when they were cocultured with MCF7 cells, indicating the enhanced migration of NIH3T3 cells that occurs after coculture with cancer cells.

Several studies pointed out that the conditioned medium from the cancer cells enhances the migration of fibroblast cells 22, 23. The effect of MCF7 or MDA‐MB 231 conditioned medium on the migration of fibroblasts was examined. The migration of fibroblasts was observed in the presence of 1% conditioned medium obtained from culturing MCF7 and MDA‐MB 231 cells for 48 h. As an alternate method, we also used the transwell migration assay to analyze the enhanced migration of the fibroblast in the presence of MCF7 cells. The MCF7 cells were cultured on upper chamber and placed onto lower chamber containing cultured NIH3T3 cells. After 24 h, the NIH3T3 cells were scratched and cultured in medium containing 1% serum. By means of both methods, no significant difference was observed in the migration of fibroblast (Fig. S1A and B). These results indicate the possibility that cell‐to‐cell contact might be essential for enhanced migration.

In order to examine whether the phenomenon of enhanced migration of fibroblasts is restricted only to MCF7 cells, other cancer cells were also used for coculturing. Highly invasive cancer cells are difficult to use in wound healing coculture assay for observing the enhanced migration of fibroblasts, as such cells migrate faster compared to fibroblasts. Therefore, the HeLa human cervical cancer cells and the A549 human lung cancer cells showing slow migration compared to NIH3T3 fibroblasts were selected for wound healing coculture assay (Fig. S2A). These three cancer cells did not migrate significantly when they were cultured alone. In contrast, when NIH3T3 cells were cocultured with HeLa and A549 cancer cells at ratio (5:1), same ratio used for coculturing of MCF7 cancer cells, significant enhanced migration of NIH3T3 fibroblasts was observed when compared to migration of NIH3T3 alone at 24 h (Fig. S2B and C). These results indicate that the enhanced migration of fibroblasts is not restricted only to MCF7 cells but can also be seen in other cancer cell lines as HeLa and A549 cells.

Although NIH3T3 normal mouse embryonic fibroblast is easy to culture and is suitable to use in high‐throughput wound healing assay, it possesses some characteristics such as unlimited ability of proliferation which makes it different from normal human fibroblasts cells. In order to confirm the phenomenon of enhanced migration of fibroblast is not due to characteristic of one particular fibroblast cells, the human lung fibroblast cell WI‐38 was used for coculturing with MCF7 cells. Using wound healing coculture assay, significant enhanced migration of WI‐38 cells was observed when cocultured with MCF7 cells at 24 h after scratched. These results thus show that the phenomenon of enhanced migration of fibroblast is not limited to NIH3T3 cells (Fig. S3A and B).

### Increased expression of *β*‐arrestin1 in cocultured cells

**Figure 2:**
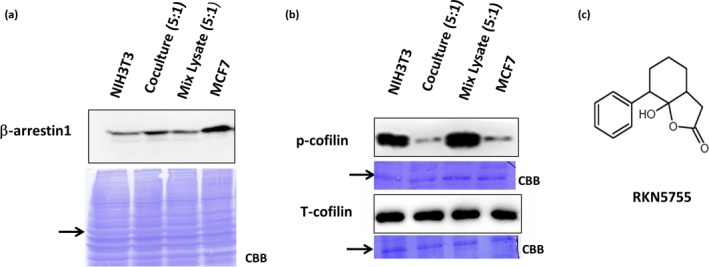
Expression of *β*‐arrestin1 and p‐cofilin during MCF7‐induced migration of NIH3T3 cells. (A) *β*‐arrestin1 expression in NIH3T3 fibroblasts, NIH3T3 cocultured cells (5:1), and MCF7 cells cultured for 48 h were detected by Western blotting. To prepare the mix lysate, cell lysates from wells of NIH3T3 alone and MCF7 alone were mixed at 5:1 ratio and quantified. Cell lysates of equal amount of protein were loaded and analyzed with Western blotting. Coomassie staining of the blot was used as a control for protein loading. Arrows indicate the position of the *β*‐arrestin1 protein. (B) Ser^3^ phosphorylation levels of cofilin (p‐cofilin; inactivated form) and total cofilin (cofilin) in NIH3T3 fibroblasts, NIH3T3 cocultured cells (5:1), and MCF7 cells cultured for 48 h were detected by Western blotting. NIH3T3 and MCF7 cell lysates were mixed at a similar ratio (5:1) to that of the coculture and used as a control. Coomassie staining of the blot was used as a loading control. Arrows indicate the position of p‐cofilin and cofilin protein. (C) Chemical structure of RKN5755.

As high expression of *β*‐arrestin1 has been reported in CAFs 10, we examined its expression in NIH3T3 cells in our coculture system. Although significant levels of *β*‐arrestin1 expression were detected in NIH3T3 cells, higher expression was detected in MCF7 cells (Fig. 2A). When MCF7 cells were cocultured with NIH3T3 cells, the expression was significantly enhanced in the coculture (Fig. 2A, coculture). This increased level of signal intensity was not caused by the high expression level of *β*‐arrestin1 in MCF7 cells, because it was significantly higher than in the mixed lysates of NIH3T3 and MCF7 cells cultured separately (Fig. 2A, mixed lysates).

### Enhanced dephosphorylation and activation of cofilin in coculture

Cofilin is known to be a downstream protein of *β*‐arrestin1. Binding of *β*‐arrestin1 to cofilin induces dephosphorylation at the third serine residue (Ser3) and activation of cofilin, which plays an important role in cell migration 16. As shown in Figure 2B (upper panel), Ser3 phosphorylation of cofilin (p‐cofilin; inactivated form) was higher in NIH3T3 cells compared to that in MCF7 cells, which express a high level of *β*‐arrestin1. The high level of p‐cofilin in NIH3T3 cells was dramatically decreased after 24 h of coculture with MCF7 cells, indicating that cofilin activation was induced after coculture (Fig. 2B, coculture). We confirmed that this decrease did not occur when separately cultured cell lysates were mixed together (mixed lysates).

### Screening of *β*‐arrestin1 small‐molecule ligands

To identify compounds that inhibit the enhanced migration of NIH3T3 cells that occurs in the presence of MCF7 cells, we attempted to isolate ligands of *β*‐arrestin1. GST‐tagged *β*‐arrestin1 protein was purified and used for the screening of small molecule ligands, using the RIKEN NPDepo chemical array. After screening 29,707 compounds, 377 compounds were identified as hit compounds (Fig. S4A).

These hit compounds were examined for their ability to inhibit the enhanced migration that occurs in the coculture system. In repeated analyses of the wound healing assay using cocultured cells, three compounds reproducibly inhibited the enhanced migration of the cocultured NIH3T3 cells at 1 *μ*g/mL (Fig. S4B and C). Among these three compounds, one compound inhibited the migration of NIH3T3 cells and the other one inhibited the migration of MCF7 cells; therefore, only one remaining compound, RKN5755 (Fig. 2C), was selected for further analyses (Fig. S4D).

### RKN5755 inhibits enhanced migration of cocultured NIH3T3 cells in a dose‐dependent manner

**Figure 3:**
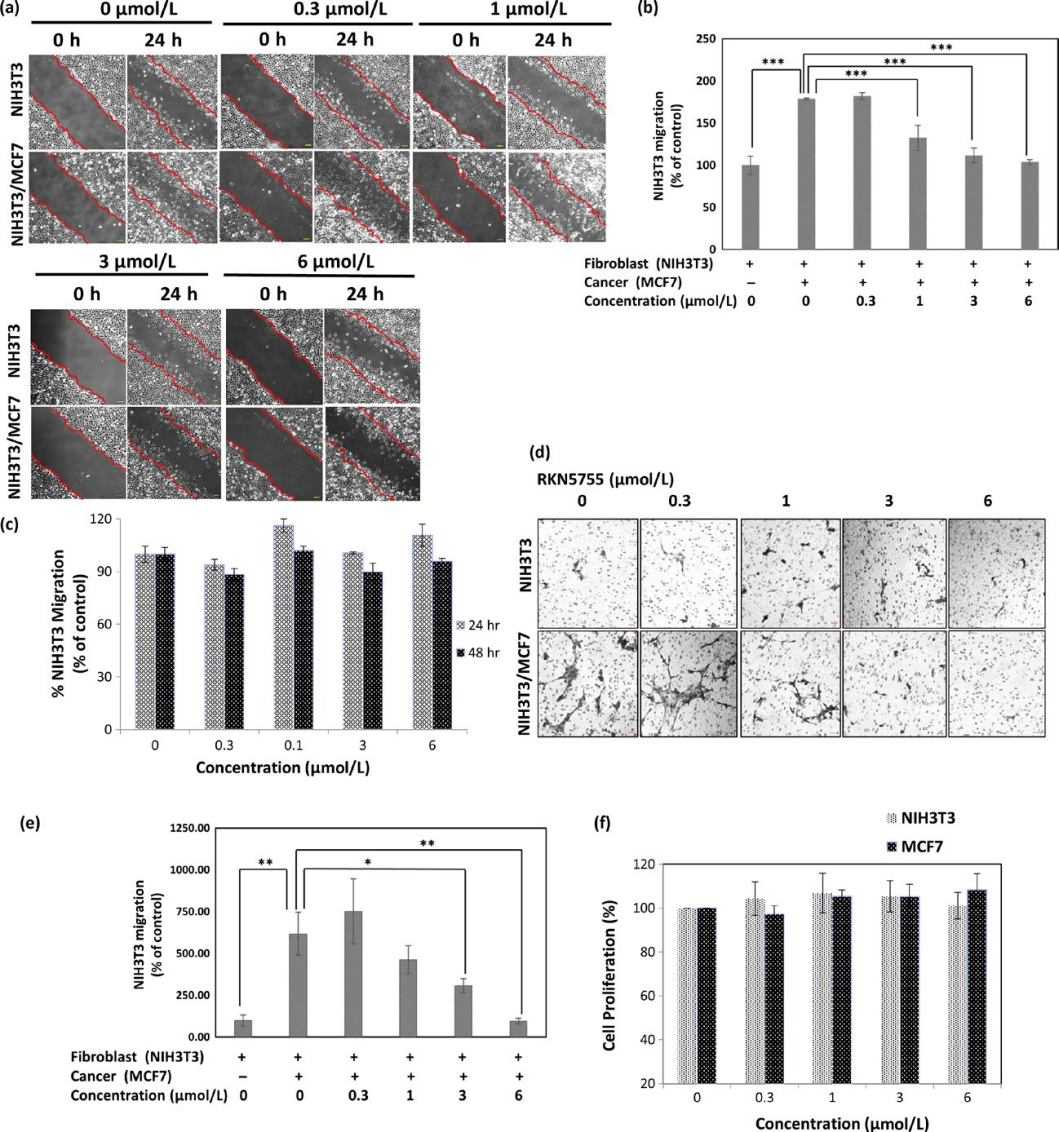
RKN5755 suppresses enhanced migration of NIH3T3 cells cocultured with MCF7 cells. (A) NIH3T3 cells alone or NIH3T3 cells cocultured with MCF7 were seeded overnight in 6‐well plates in 10% serum. On the next day, the cells were scratched, treated with different concentrations of RKN5755, and cultured in medium containing 1% FCS. The edge of the wound at the 0‐h time point is marked with a red outline and was overlaid with other images taken at 24 h. (B) The cell migration percentage for fibroblasts and cocultured cells was calculated using ImageJ. The graph shows the percentage of fibroblast migration in coculture relative to the control (fibroblast alone) at 24 h and was plotted against the RKN5755 concentration used for treatment. For the control, there was a significant difference in the migration of cocultured fibroblasts when compared to fibroblasts alone at 24 h (*n* = 3, *** *P* < 0.0005). RKN5755 treatments of 3 *μ*mol/L or 6 *μ*mol/L showed a significant decrease in NIH3T3 migration when cocultured with MCF7 (*n* = 3, *** *P* < 0.0005). (C**)** NIH3T3 cells were seeded overnight in 6‐well plates in 10% serum. On the next day, the cells were scratched and treated with different concentrations of RKN5755 and cultured in medium containing 1% FCS. The fibroblast migration percentage was calculated using ImageJ software. The graph indicates the fibroblast migration percentage occurring in coculture, relative to the control at 48 h, and was plotted against the concentration of RKN5755. RKN5755 treatment, even at the high dose of 6 *μ*mol/L for 48 h, did not inhibit the migration of NIH3T3 cells. (D) NIH3T3 cells and cocultured NIH3T3 cells were grown without serum in the upper chamber and were allowed to migrate toward the bottom chamber containing 10% serum. Different concentrations of RKN5755 were added to both the upper and bottom chambers. The membrane was stained with crystal violet after 24 h of migration, and images of the three different fields were captured with an inverted microscope. (E) The percentage of fibroblasts that migrated in coculture or when cultured alone in D was quantified using ImageJ. The graph shows the percentage of fibroblasts migrating in coculture compared to fibroblast cultured alone and was plotted against different doses of RKN5755. In the control (without compound), there was a significant difference in the migration of cocultured fibroblasts compared to that by fibroblasts alone at 24 h (*n* = 3, ** *P* < 0.005). Treatment of RKN5755 at 3 *μ*mol/L or 6 *μ*mol/L showed a significant decrease in the migration of NIH3T3 cells cocultured in the presence of MCF7 cells (*n* = 3, * *P* < 0.05, ** *P* < 0.005). (F) Cell proliferation using the WST‐8 reagent was carried out in NIH3T3 and MCF7 cells cultured in 1% serum for 48 h. RKN5755 treatment up to 6 *μ*mol/L did not inhibit the growth of NIH3T3 and MCF7 cells.

The inhibition of cocultured cell‐enhanced migration was examined in the presence of various concentrations of RKN5755 (Fig. 3A and B). Although the migration of NIH3T3 cells alone was not affected by RKN5755 (Fig. 3A, upper panels; Fig. 3C), significant inhibition of cocultured cells enhanced migration was observed in the presence of 3 and 6 *μ*mol/L RKN5755 (Fig. 3A, lower panels; Fig. 3B).

A similar effect of RKN5755 on enhanced migration was observed using the transwell assay (Fig. 3D and E). In the presence of up to 6 *μ*mol/L RKN5755, the migration of NIH3T3 cells through the filter was not affected, although the number of migrated cells was very small in the absence of MCF7 (Fig. 3D upper panels; Fig. 3E). In contrast, a significant inhibition of enhanced migration was detected in the presence of 3 and 6 *μ*mol/L of RKN5755 (Fig. 3D, lower panels; Fig. 3E). At higher concentration of RKN5755, the migration was inhibited in a dose‐dependent manner (Fig. 3D, lower panels; Fig. 3E). We confirmed that the inhibition of enhanced migration was not caused by the cytotoxic effect of RKN5755, since up to a 6 *μ*mol/L concentration of RKN5755 did not have any inhibitory effect on the growth of NIH3T3 or MCF7 cells (Fig. 3F). We also checked the effect of RKN5755 on enhanced migration of human WI‐38 fibroblast cells in the presence of MCF7 cancer cells. Significant inhibition of enhanced migration of human WI‐38 fibroblast cocultured cells was observed at 6 *μ*mol/L concentration of RKN5755. Thus, RKN5755 might be acting specifically on fibroblast‐activated cancer cells. However, in future, more detail investigation is required. (Fig. S6A and B).

In order to determine whether RKN5755 is inhibiting *β*‐arrestin1‐induced fibroblasts or MCF7 cells, we treated NIH3T3 cells and MCF7 cells with RKN5755 at 6 *μ*mol/L concentration for 24 h, separately. RKN5755‐treated NIH3T3 cells was co‐cultured with control MCF7 cells (without prior compound treatment) and control NIH3T3 cells (without prior compound treatment) was co‐cultured with RKN5755 treated MCF7 cancer cells, then scratched and migration was observed for 24 h. Significant decrease in the enhanced migration of fibroblast was observed when treated with NIH3T3 cells but not with MCF7 cells after 24 h of coculturing. Thus, the compound RKN5755 might be specifically acting on fibroblasts (Fig. S5).

### RKN5755 interacts with *β*‐arrestin1 and inhibits the dephosphorylation of cofilin

**Figure 4:**
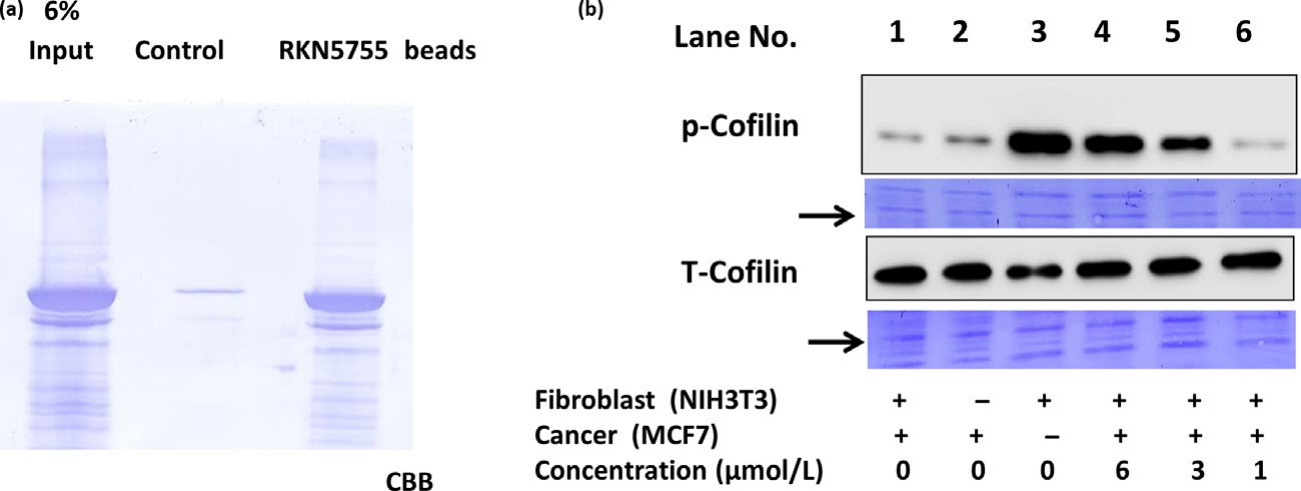
RKN5755 interacts with *β*‐arrestin1 and inhibits the dephosphorylation of cofilin. (A) Purified GST‐*β*‐arrestin1 from bacterial lysate was incubated with control and RKN5755 beads for 3 h. The beads were then washed with binding buffer and eluted with SDS‐PAGE sample buffer. Purified *β*‐arrestin1 (6% of input) was loaded as a control. (B) The Ser^3^ phosphorylation levels of cofilin (p‐cofilin; inactivated form) and total cofilin (cofilin) in NIH3T3 fibroblasts, NIH3T3 cocultured cells (5:1), and MCF7 cells cultured and treated with RKN5755 at different concentrations for 24 h were detected by Western blotting. Coomassie staining of the blot was used as a control for protein loading. Arrows indicate the position of p‐cofilin and cofilin proteins.

As RKN5755 is a hit compound identified by a chemical array for *β*‐arrestin1, it was predicted to interact with *β*‐arrestin1. To confirm this, we fixed RKN5755 onto agarose beads and examined the binding of *β*‐arrestin1 to the beads. When purified, GST‐*β*‐arrestin1 proteins from bacterial lysates were pulled down and analyzed by SDS‐PAGE and *β*‐arrestin1 was pulled down only with RKN5755‐bound beads but not with control beads unbound to *β*‐arrestin1, indicating the physical interaction of RKN5755 and *β*‐arrestin1 (Fig. 4A).

The above results suggest that the direct interaction of RKN5755 to *β*‐arrestin1 may inhibit cofilin activation. Consistent with the previous results, the level of p‐cofilin (inactivated form) was high in NIH3T3 cells, but was dramatically reduced in the presence of cocultured MCF7 (Fig. 4B, lane 1). However, in the presence of 1 *μ*mol/L RKN5755, the level of p‐cofilin was significantly increased, indicating that RKN5755 inhibits the activation of cofilin (Fig. 4B, lane 6). At higher concentrations, p‐cofilin levels increased in a dose‐dependent manner. At 6 *μ*mol/L, level of p‐cofilin was similar to that observed in the absence of cocultured MCF7 cells, indicating the complete inhibition of the enhanced effect resulting from coculture with MCF7 (Fig. 4B, lane 4 and 3). Taken together, our results show that *β*‐arrestin1 plays an important role in the enhanced migration of NIH3T3 cells that occurs in the presence of cocultured MCF7 cells through the dephosphorylation (activation) of cofilin. Moreover, we found that RKN5755 directly interacts with *β*‐arrestin1 and inhibits the activation of cofilin.

## Discussion

In this study, we demonstrate that NIH3T3 fibroblasts migrate faster when cocultured with MCF7 breast cancer cells. Most current research has focused on the enhanced migration of breast cancer cells occurring when cocultured with fibroblasts. These studies identified that TGF‐*β* secretion by fibroblasts, under the influence of cancer cells, promotes the enhanced migration of breast cancer cells 24. Another report suggested that cross‐talk between fibroblasts and cancer cells by SDF1‐CXCR4 signaling facilitates cancer cell migration 25. Using an established wound healing coculture assay and the transwell coculture system, we also showed that cancer cells promote the enhanced migration of fibroblasts. Significant enhanced migration was observed when fibroblasts were directly cocultured with cancer cells at a specific ratio of 5:1. Mouse embryonic fibroblasts NIH3T3 and low‐invasive breast cancer cells MCF7 were selected for coculturing. As MDA‐MB 231 cells migrate faster when compared to NIH3T3 cells, MCF7 cells were selected. We observed similar phenomenon of enhanced migration when other low‐invasive cancer cells were used for wound healing coculture assay. Conditioned medium obtained from MCF7 and MDA‐MB 231 cells did not induce enhanced migration of fibroblasts. Therefore, this indicates that direct cell‐to‐cell contact between fibroblasts and cancer cells might be required for showing such an effect of enhanced migration by fibroblasts. Several researchers have studied the heterotypic cell adhesion junctions between different cadherin pairs 26, 27, 28. Their observation supports the recent work which revealed that heterotypic cell adhesion junction interaction between fibroblasts and cancer cells is important for CAF‐guided cancer cell invasion 26.

Additionally, we report the possible role of *β*‐arrestin1 in enhancing fibroblast migration when cocultured with cancer cells by modulating cofilin actin‐severing activity. Our study also suggests that fibroblast activation can be specifically targeted using small molecules and that this can be examined by high‐throughput approaches in vitro using a coculture system.

Other reports suggest that cancer cells can also activate fibroblasts and can modulate functional morphological change by enhancing fibroblasts migration 28. We examined *β*‐arrestin1 levels and the status of its binding protein cofilin in NIH3T3 cells cocultured with MCF7 cells and observed the increased expression of *β*‐arrestin1 as well as decreased phosphorylation (inactive) levels of cofilin. *β*‐arrestins are adaptor molecules that bind to a number of 7‐transmembrane (7TM) domain receptors and also bind to signaling molecules involved in chemotaxis and cell migration. Protease‐activated receptor‐2 is one such 7TM‐receptor, which recruits *β*‐arrestins for cell migration. Cell migration requires pseudopodia formation and extension, which is carried out by regulation of actin polymerization and depolymerization via the cofilin pathway. *β*‐arrestin1 helps in the localization of active cofilin to the leading edge of the migrating cell in response to PAR‐2 receptor activation 29. A few studies found elevated expression of the PAR‐2 receptor in the stromal region of the tumor as well as evaluated the role of tumor growth factor *β* (TGF‐*β*) in increasing PAR‐2 expression in fibroblasts 30, 31. Thus, increased *β*‐arrestin1 expression in NIH3T3 cells cocultured with MCF7 might be due to the increased expression of PAR‐2 receptor in response to growth factors secreted by cancer cells. Increased expression of *β*‐arrestin1 promotes dephosphorylation of cofilin, thereby causing enhanced fibroblast migration. Targeting the *β*‐arrestin1–cofilin signaling pathway might help in inhibiting the activation of fibroblasts involved in cancer metastasis.

As CAFs play an important role in cancer metastasis, it is very important to identify small‐molecule inhibitors which could eliminate the effects of CAFs. To date, immunotherapy has been studied as a treatment option for targeting CAFs in cancer therapy 32. We attempted to identify novel chemical inhibitors of the CAF activator to treat cancer metastasis. Targeting pathways dependent on *β*‐arrestin1 for treating CAFs are quite complicated as *β*‐arrestin1 binds to many (7TM)‐receptors as well as many downstream signaling proteins. Thus, we used chemical array screening to identify small‐molecule ligands of *β*‐arrestin1 and used a cell migration wound healing assay to target *β*‐arrestin1 signaling pathways involved in chemotaxis and cell migration. We found that compound RKN5755 binds to *β*‐arrestin1 and is capable of restoring the cofilin phosphorylation level in fibroblasts cocultured with cancer cells. This indicates that compound RKN5755 interferes with the *β*‐arrestin1–cofilin scaffolding pathway, thus inhibiting the enhanced migration of fibroblasts activated by cancer cells.

Our results indicate that, fibroblasts activated by cancer cells show enhanced migration and that this property can be targeted by small molecules. Although the exact mechanism that leads to activation of fibroblast by cancer cells is not fully understood, target‐based screening using chemical array analysis might give us insights into the role of proteins involved in the activation of fibroblasts. Understanding the mechanism of activation may help further the development of targeted therapies against CAFs, which occupy a major portion of the tumor microenvironment. Thus, a combination of conventional therapy with a CAF‐directed therapy might lead to complete treatment of cancer metastasis.

## Conflict of Interest

The authors declare no conflict of interest.

## Supporting information


**Figure S1**. Effect of cancer condition medium and culturing cancer cells separately using transwell on fibroblast migration.
**Figure S2.** Migration of NIH3T3 fibroblast cells when co‐cultured with other cancer cells.
**Figure S3.** WI‐38 cells co‐cultured with MCF7^GFP^ cells display enhanced migration activity compared to culture of WI‐38 cells alone.
**Figure S4.** Chemical array analysis and screening using wound healing co‐culture assay.
**Figure S5.** NIH3T3 cells pre‐treated with RKN5755 display decreased migration activity compared to NIH3T3 cells without treatment.
**Figure S6.** RKN5755 suppresses enhanced migration of WI‐38 cells co‐cultured with MCF7 cells.
**Figure S7.** Protein BLAST results for *β*‐arrestin1 protein sequence from human and mice.
