# Supplementary material for: Identification of a small‐molecule ligand of β‐arrestin1 as an inhibitor of stromal fibroblast cell migration accelerated by cancer cells
Source: Cancer Med. 2018 Jan 29;7(3):883–93. doi: 10.1002/cam4.1339 (PMC5852355; doi:10.1002/cam4.1339)
Supplement: Supplementary file 1 — Figure S1. Effect of cancer condition medium and culturing cancer cells separately using transwell on fibroblast migration. Figure S2. Migration of NIH3T3 fibroblast cells when co‐cultured with other cancer cells. Figure S3. WI‐38 cells co‐cultured with MCF7GFP cells display enhanced migration activity compared to culture of WI‐38 cells alone. Figure S4. Chemical array analysis and screening using wound healing co‐culture assay. Figure S5. NIH3T3 cells pre‐treated with RKN5755 display decreased migration activity compared to NIH3T3 cells without treatment. Figure S6. RKN5755 suppresses enhanced migration of WI‐38 cells co‐cultured with MCF7 cells. Figure S7. Protein BLAST results for β‐arrestin1 protein sequence from human and mice. [file CAM4-7-883-s001.pdf]

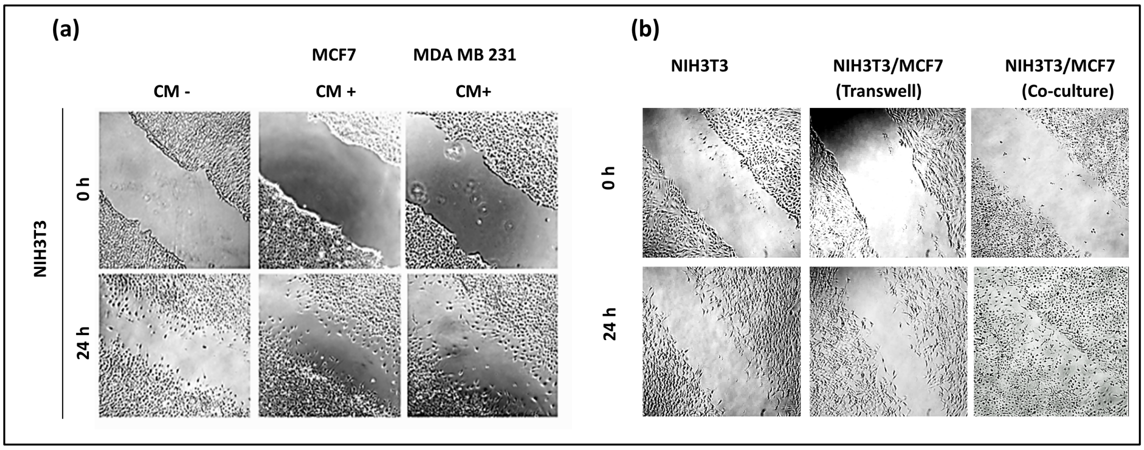

Fig S1. Effect of cancer condition medium and culturing cancer cells separately using transwell on fibroblast migration. (a) 1% conditioned medium was obtaining from culturing MCF7 and MDA MB 231 cancer cells in DMEM containing 10% FBS for 48 hours. NIH3T3 alone were seeded in DMEM containing 10% FBS in 24-well plates. After overnight culture, these cells were scratched and medium was replaced with 1% conditioned medium. The migration was observed at 24 hours. There was no difference in migration of NIH3T3 in the presence of conditioned medium when compared to control. (b) MCF7 cells and NIH3T3 cells were cultured separately using transwell chamber. The MCF7 cells were cultured on upper chamber and placed onto lower chamber containing cultured NIH3T3 cells in 24-well plates. After overnight incubation, the cells were scratched and cultured in medium containing 1% serum. The migration was observed at 24 hours. There was no difference in migration of NIH3T3 cells in the presence of MCF7 cells when compared to control.

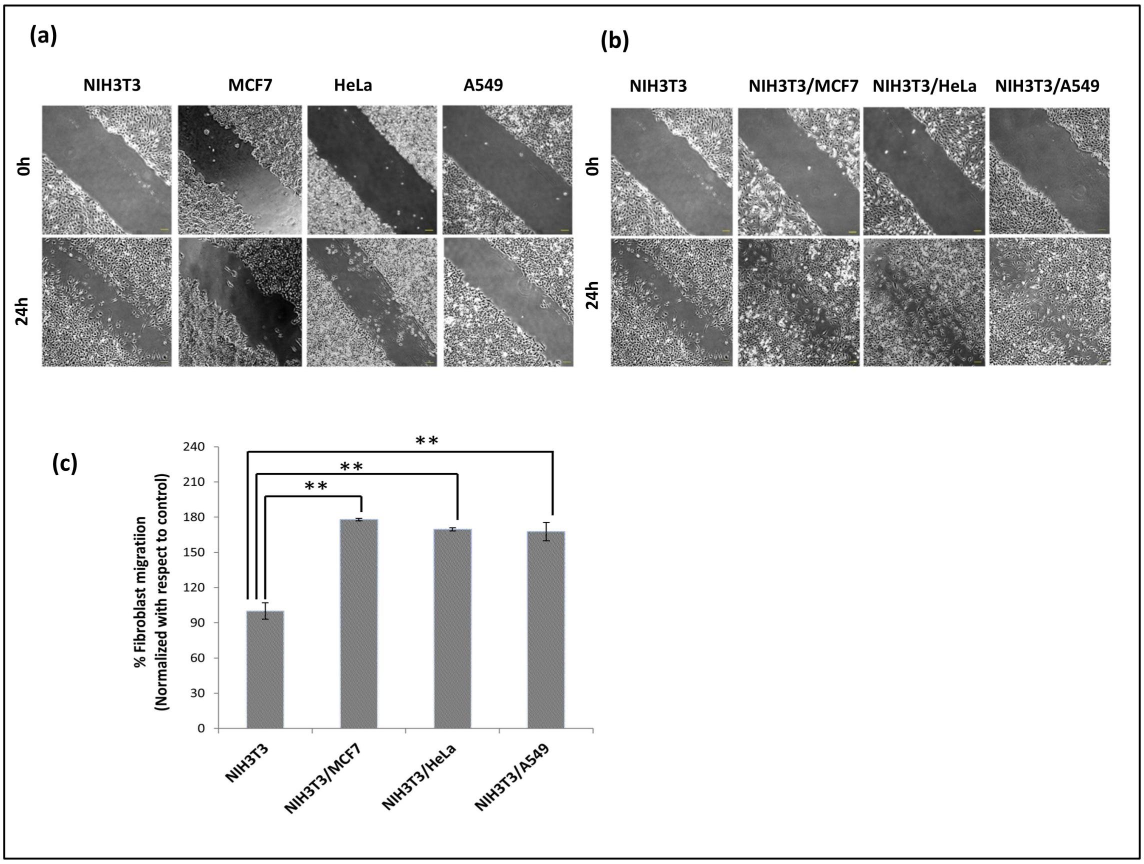

Fig S2. Migration of NIH3T3 fibroblast cells when co-cultured with other cancer cells. (a) NIH3T3 fibroblast cells, MCF7 breast cancer cells, HeLa cervical cancer cells and A549 lung cancer cells were seeded in 6-well plates, scratched and cultured in medium containing 1% FCS. The cell migration was observed at 24 hours. (b) NIH3T3 cells alone, NIH3T3 cells co-cultured with MCF7 cells, HeLa cells and A549 cells seeded in 6-well plates, scratched and cultured in medium containing 1% FCS. The cell migration was observed at 24 hours. (c) The cell migration percentage for fibroblasts and co-cultured fibroblasts was quantified using ImageJ. There was a significant difference in the migration of co-cultured fibroblasts when compared to culture of fibroblasts alone at 24 hours (n = 3, $^{**}P < 0.005$).

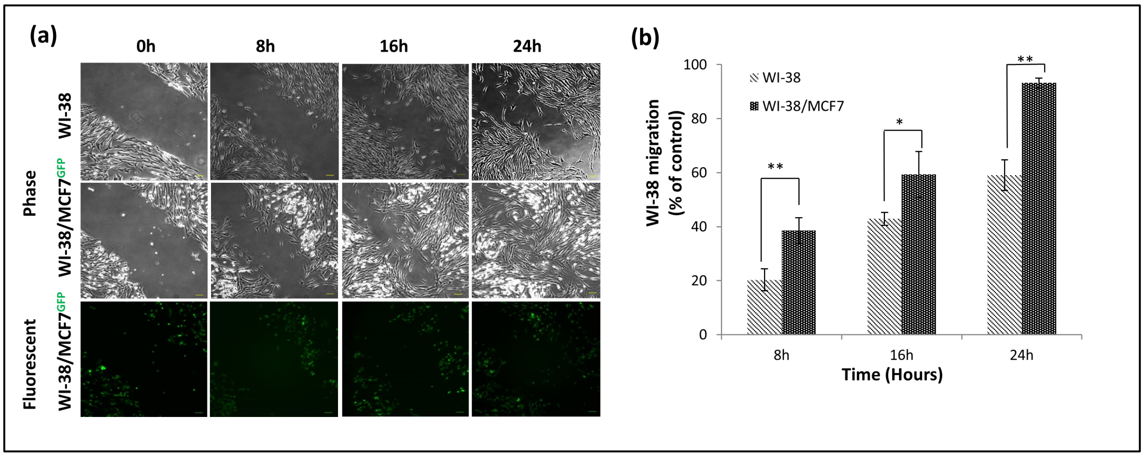

Fig S3. WI-38 cells co-cultured with MCF7^{GFP} cells display enhanced migration activity compared to culture of WI-38 cells alone. (a) WI-38 cells alone and WI-38 cells co-cultured with MCF7 were seeded in 6-well plates, scratched, and cultured in medium containing 1% FCS. Cell migration was observed at different time points until 24 hours. Images of MCF7 tagged with GFP in co-culture were taken by fluorescent and light microscopy. (b) The cell migration percentage for fibroblasts and co-cultured cells at different time points was quantified using ImageJ software. There was a significant difference in the migration of co-cultured fibroblasts when compared to culture of fibroblasts alone at 8 hours, 16 hours and 24 hours (n = 3,*$P < 0.05$,**$P < 0.005$, ***$P < 0.0005$).

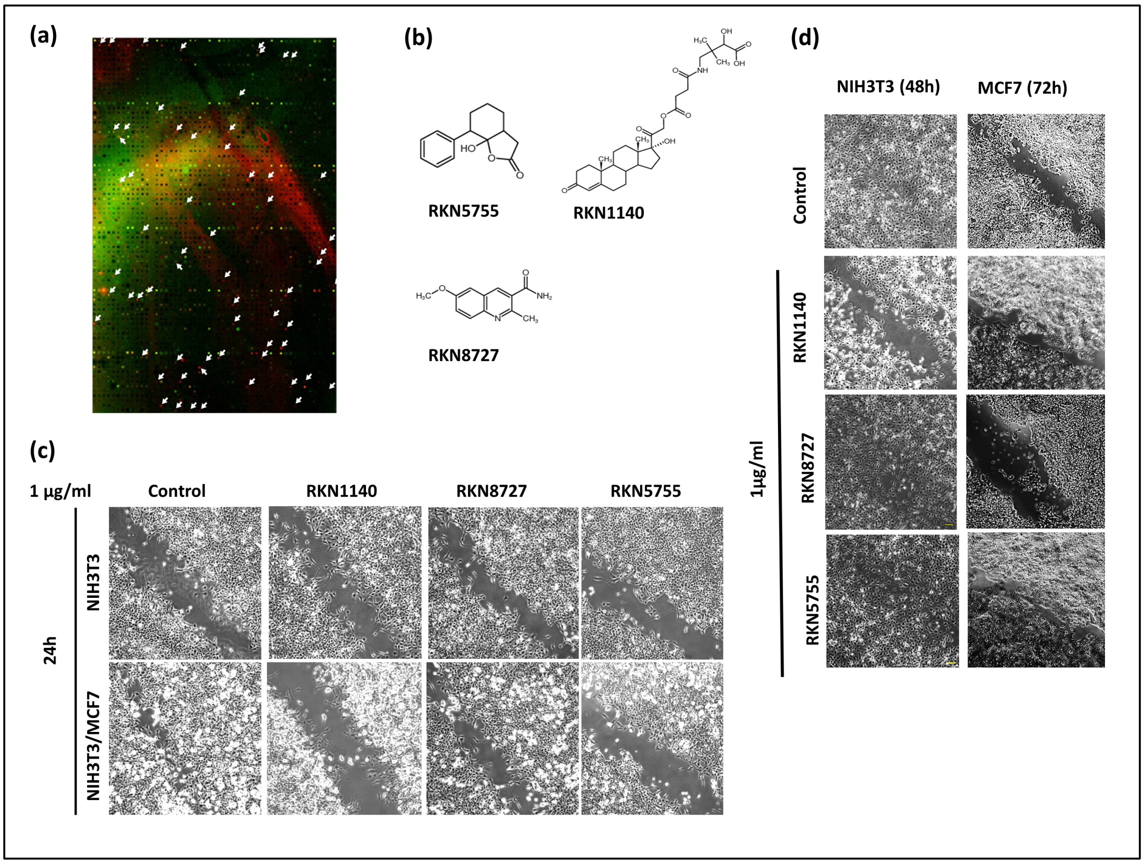

Fig S4. Chemical array analysis and screening using wound healing co-culture assay. (a) Representative image of chemical array. The chemical arrays were probed sequentially with GST-tagged β-arrestin1 protein or GST protein, anti-GST antibody, and a Cy5-labeled secondary antibody, and then scanned with a microarray scanner. The fluorescent images of 2 slides, with GST-tagged β-arrestin1 and GST, were tinted red and green, respectively, and merged into a composite image. Candidate compounds are indicated with white arrows. (b) Chemical structure of RKN5755, RKN1140 and RKN8727. (c) NIH3T3 cells alone and NIH3T3 cells co-cultured with MCF7 cells were seeded in 6-well plates, scratched and cultured in medium containing 1% FCS. The cells were treated with 1ug/ml concentration of respective compound. The migration was observed at 24 hours. (d) NIH3T3 cells and MCF7 cells alone were seeded in 6-well plates, scratched and cultured in medium containing 1% FCS. The cells were treated with 1ug/ml concentration of respective compound. The migration was observed till 48 hours and 72 hours respectively.

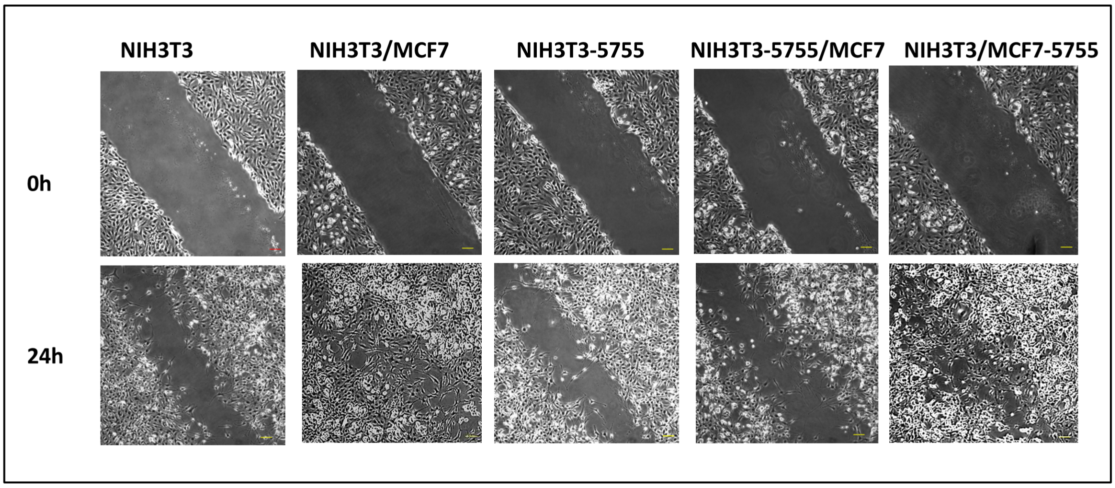

Fig S5. NIH3T3 cells pre-treated with RKN5755 display decreased migration activity compared to NIH3T3 cells without treatment. NIH3T3 cells were treated with 6 µM concentration of RKN5755 for 24 hours. Next day, the RKN5755 treated NIH3T3 cells (NIH3T3-5755), NIH3T3-5755 co-cultured with MCF7 cells, NIH3T3 cells alone and NIH3T3 cells co-cultured with MCF7 cells were seeded, scratched and cultured in medium containing 1% FCS. The migration was observed at 24 hours. MCF7 cells were treated with 6 µM concentration of RKN5755 for 24 hours. Next day, the RKN5755 treated MCF7 cells (MCF7-5755) and NIH3T3 co-cultured with MCF7-5755 cells were seeded, scratched and cultured in medium containing 1% FCS. The migration was observed at 24 hours

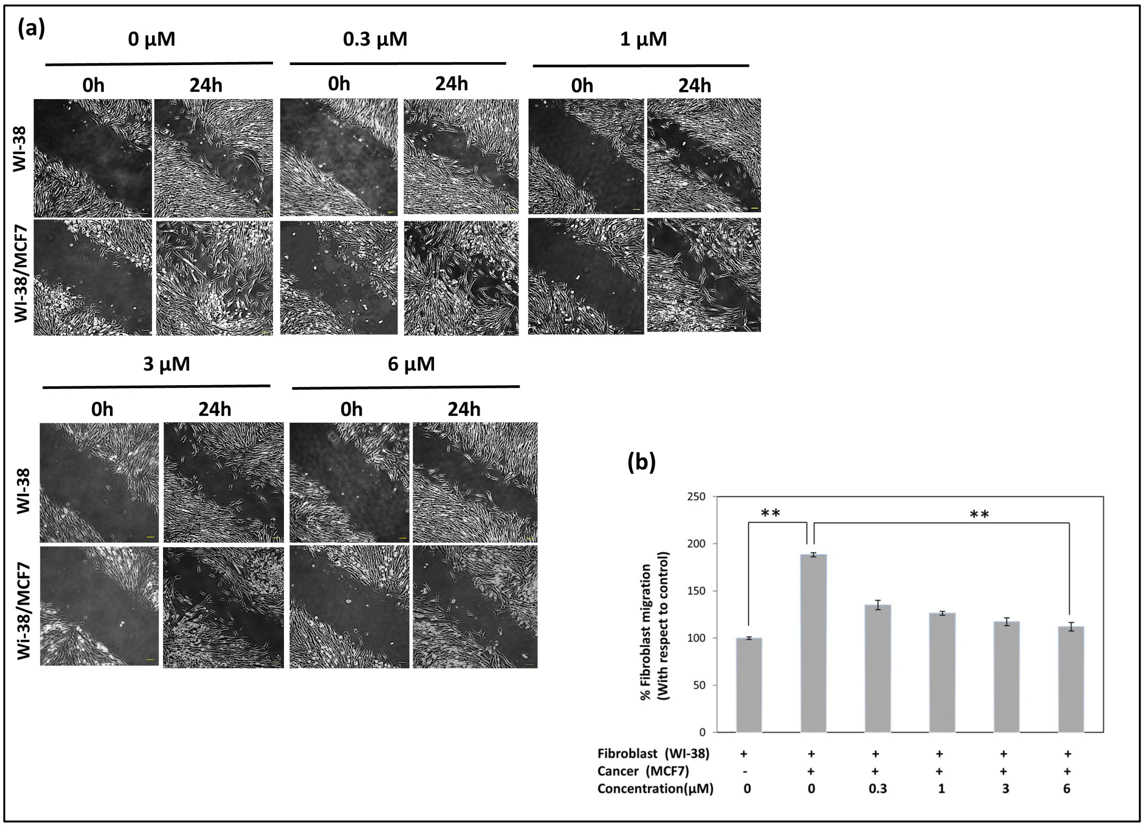

Fig S6. RKN5755 suppresses enhanced migration of WI-38 cells co-cultured with MCF7 cells. (a) WI-38 cells alone or WI-38 cells co-cultured with MCF7 were seeded overnight in 6-well plates in 10% serum. On the next day, the cells were scratched, treated with different concentrations of RKN5755, and cultured in medium containing 1% FCS. The migration was observed at 24 hours. (b) The cell migration percentage for fibroblasts and co-cultured cells was calculated using ImageJ. The graph shows the percentage of fibroblast migration in co-culture relative to the control (fibroblast alone) at 24 hours and was plotted against the RKN5755 concentration used for treatment. For the control, there was a significant difference in the migration of co-cultured fibroblasts when compared to fibroblasts alone at 24 hours ($n = 3$,$^{**}P < 0.005$). RKN5755 treatments of 3 μM or 6 μM showed a significant decrease in WI-38 migration when co-cultured with MCF7 ($n = 3$,$^{**}P < 0.005$).

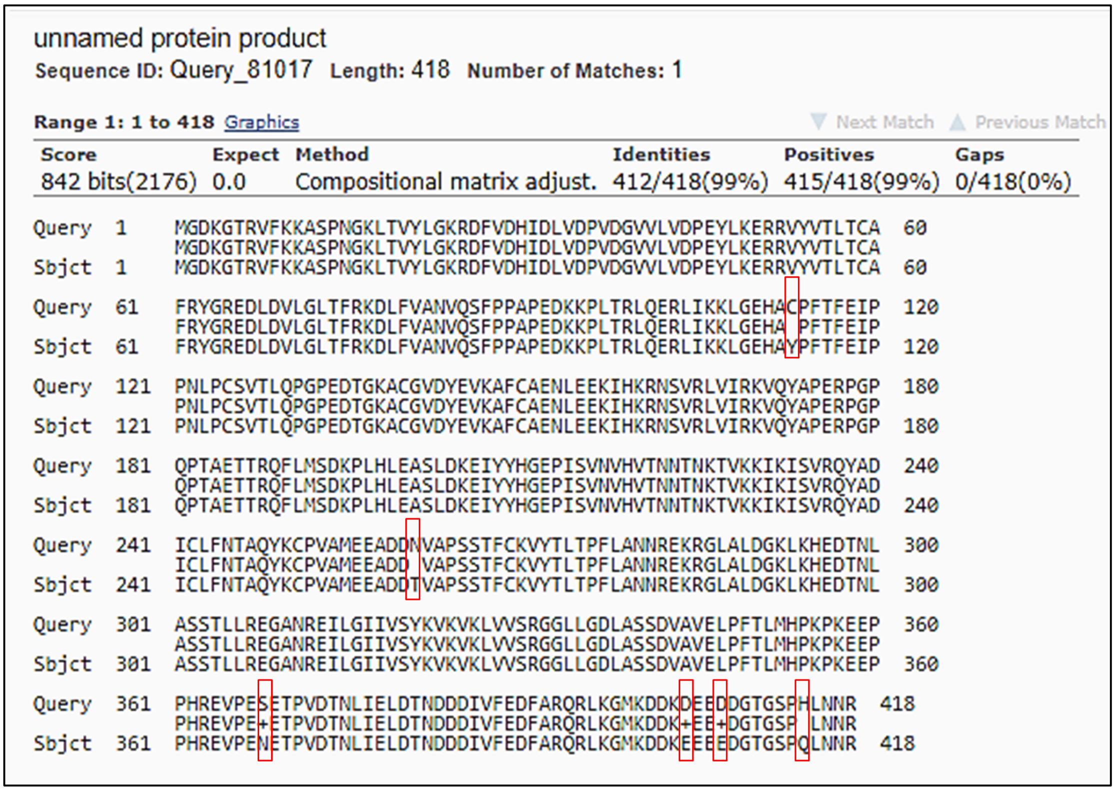
unnamed protein product

Sequence ID: Query_81017 Length: 418 Number of Matches: 1

Range 1: 1 to 418 Graphics ▼ Next Match ▲ Previous Match

Score	Expect	Method	Identities	Positives	Gaps
842 bits(2176)	0.0	Compositional matrix adjust.	412/418(99%)	415/418(99%)	0/418(0%)

```
Query  1    MGDKGTRVFKKASPNGKLTVYLGKRDFVDHIDLVDPVDGVVLVDPEYLKERRVYVTLTCA  60
            MGDKGTRVFKKASPNGKLTVYLGKRDFVDHIDLVDPVDGVVLVDPEYLKERRVYVTLTCA
Sbjct  1    MGDKGTRVFKKASPNGKLTVYLGKRDFVDHIDLVDPVDGVVLVDPEYLKERRVYVTLTCA  60

Query  61   FRYGREDLDVLGLTFRKDLFVANVQSFPPAPEDKKPLTRLQERLIKKLGEHACPFTFEIP  120
            FRYGREDLDVLGLTFRKDLFVANVQSFPPAPEDKKPLTRLQERLIKKLGEHA PFTFEIP
Sbjct  61   FRYGREDLDVLGLTFRKDLFVANVQSFPPAPEDKKPLTRLQERLIKKLGEHAYPFTFEIP  120

Query  121  PNLPCSVTLQPGPEDTGKACGVDYEVKAFCAENLEEKIHKRNSVRLVIRKVQYAPERPGP  180
            PNLPCSVTLQPGPEDTGKACGVDYEVKAFCAENLEEKIHKRNSVRLVIRKVQYAPERPGP
Sbjct  121  PNLPCSVTLQPGPEDTGKACGVDYEVKAFCAENLEEKIHKRNSVRLVIRKVQYAPERPGP  180

Query  181  QPTAETTRQFLMSDKPLHLEASLDKEIYYHGEPISVNVHVTNNTNKTVKKIKISVRQYAD  240
            QPTAETTRQFLMSDKPLHLEASLDKEIYYHGEPISVNVHVTNNTNKTVKKIKISVRQYAD
Sbjct  181  QPTAETTRQFLMSDKPLHLEASLDKEIYYHGEPISVNVHVTNNTNKTVKKIKISVRQYAD  240

Query  241  ICLFNTAQYKCPVAMEEADDNVAPSSTFCKVYTLTPFLANNREKRGLALDGKLKHEDTNL  300
            ICLFNTAQYKCPVAMEEADD VAPSSTFCKVYTLTPFLANNREKRGLALDGKLKHEDTNL
Sbjct  241  ICLFNTAQYKCPVAMEEADDTVAPSSTFCKVYTLTPFLANNREKRGLALDGKLKHEDTNL  300

Query  301  ASSTLLREGANREILGIIVSYKVKVKLVVSRGGLLGDLASSDVAVELPFTLMHPKPKEEP  360
            ASSTLLREGANREILGIIVSYKVKVKLVVSRGGLLGDLASSDVAVELPFTLMHPKPKEEP
Sbjct  301  ASSTLLREGANREILGIIVSYKVKVKLVVSRGGLLGDLASSDVAVELPFTLMHPKPKEEP  360

Query  361  PHREVPESETPVDTNLIELDTNDDDIVFEDFARQRLKGMKDDKDEEDDGTGSPHLNNR  418
            PHREVPE+ETPVDTNLIELDTNDDDIVFEDFARQRLKGMKDDK+EE+DGTGSP LNNR
Sbjct  361  PHREVPENETPVDTNLIELDTNDDDIVFEDFARQRLKGMKDDKEEEEDGTGSPQLNNR  418
```

Fig S7. Protein BLAST results for β-arrestin1 protein sequence from human and mice. Query = Mouse and Subject = Human.
